# Endovascular Treatment of Large or Giant Non-saccular Vertebrobasilar Aneurysms: Pipeline Embolization Devices Versus Conventional Stents

**DOI:** 10.3389/fnins.2019.01253

**Published:** 2019-11-28

**Authors:** Jiejun Wang, Luqiong Jia, Zhibin Duan, Zhongxiao Wang, Xinjian Yang, Yisen Zhang, Ming Lv

**Affiliations:** ^1^Beijing Neurosurgical Institute, Beijing Tiantan Hospital, Capital Medical University, Beijing, China; ^2^Department of Neurosurgery, Jincheng People’s Hospital, Jincheng, China

**Keywords:** endovascular treatment, pipeline embolization device, large or giant, vertebrobasilar aneurysms, conventional stenting

## Abstract

**Background:**

Endovascular treatment of large or giant non-saccular vertebrobasilar aneurysms (VBAs) by conventional stents is difficult and has unsatisfactory outcomes.

**Object:**

This study was performed to retrospectively analyze the safety and efficacy of a flow diverter in treating large and giant non-saccular VBAs.

**Methods:**

We identified 78 patients with 83 large or giant non-saccular VBAs who accepted endovascular treatment with a pipeline embolization device (PED) or conventional stent from January 2014 to June 2018. The technical details of the procedure, procedure-associated complications, angiographic outcomes, and clinical outcomes were evaluated.

**Results:**

Forty-two patients (53.8%, 42/78) with 44 aneurysms (53.0%, 44/83) underwent endovascular treatment with PEDs. Thirty-six patients (46.2%, 36/78) with 39 aneurysms (47.0%, 39/83) underwent endovascular treatment with conventional stents. The complication rate of PED group and conventional stent group was 7.1% (3/42) and 5.6% (2/36), respectively (odds ratio, 0.765; 95% confidence interval, 0.121–4.851; *P* = 0.776). During a median follow-up time of 28.8 months, the complete occlusion rate in the PED group and conventional stent group was 90.2% (37/41) and 70.3% (26/37), respectively (odds ratio, 3.913; 95% confidence interval, 1.122–13.652; *P* = 0.032).

**Conclusion:**

Endovascular treatment with a PED is a promising and safe modality for large and giant non-saccular VBAs, and the complication rate is acceptable, compared with conventional endovascular treatment.

## Introduction

Flow diversion has become an important endovascular treatment technique for intracranial aneurysms (Adeeb et al., 2018). Comparing with anterior circulation aneurysms, posterior circulation aneurysms are frequently associated with a higher incidence of morbidity and mortality (Adeeb et al., 2018). The mortality rate is highest among patients with dissecting aneurysms, followed by saccular and fusiform aneurysms, because of their association with subarachnoid hemorrhage (SAH) (Griessenauer et al., 2018). In a past report, patients with non-saccular vertebrobasilar aneurysms (VBAs) and dolichoectasia had a natural history of a high overall stroke rate of 27.5%, with a mortality rate ranging from 2 to 59% (Lopes et al., 2017). According to previous reports of treating VBAs, aneurysms located in the V4 segment and distal along the course of the basilar artery are amenable to pipeline embolization device (PED) treatment (Albuquerque et al., 2015); however, the safety and efficacy of PEDs in treating large or giant non-saccular VBAs are unclear. Therefore, the study was performed to compare the safety and efficacy of PEDs versus conventional stents in treating large or giant non-saccular VBAs.

## Materials and Methods

This is a retrospective review about our experience using PEDs and conventional stents to treat large or giant non-saccular VBAs. The protocol of this study was approved by the Beijing Tiantan hospital’s institutional review board, and performance of the study was approved by the ethics committee of Beijing Tiantan Hospital.

### Patient Population

We identified 78 patients with 83 large or giant non-saccular VBAs as confirmed by digital subtraction angiography (DSA) and/or magnetic resonance imaging (MRI) and underwent endovascular treatment at our institution from January 2014 to June 2018. Patients with traumatic aneurysms and collagen vascular disorders were excluded from our study. The following information was collected: patients’ characteristics, aneurysm characteristics, antiplatelet treatment, operational details, angiographic and clinical outcomes, and procedural-associated complications. Aneurysmal parameters were measured by DSA, except in cases of some partially thrombosed aneurysms with a mass effect, in which axial MRI was also performed. Written informed consent was obtained from each patient and their relatives.

### Antiplatelet and Anticoagulation Therapies

A dual antiplatelet treatment (75 mg of clopidogrel and 100 mg of aspirin daily) for 5 days before endovascular treatment was suitable for patients without intracranial hemorrhage. A loading dose of 300 mg of aspirin and 300 mg of clopidogrel 2 h preoperatively was suitable for patients with intracranial hemorrhage. Intraoperatively, an intravenous bolus dose of heparin (100 IU/kg) was administered, and heparinization was continued to maintain an activated clotting time of 2.5 times greater than the baseline value throughout the operation. Dual antiplatelet treatment was continued for 3 months in the PED group and 6 weeks in the control group postoperatively, and aspirin was continued for life in the PED group and 6 months in the control group to prevent formation of thrombi in the stents.

### Endovascular Procedure

In the control group, patients underwent endovascular therapy with conventional stents, such as the Enterprise stent, Solitaire stent, Neuroform stent, or LVIS stent with or without adjunctive coils. In the PED group, patients underwent endovascular therapy with pipeline stents with or without adjunctive coils. The following two factors were considered when choosing the most appropriate stent. First, conventional stents were applied as a major endovascular treatment for VBAs until the emergence of the PED, which was regarded as a feasible treatment modality for this lesion. Second, the choice of stent was at the discretion of the neurointerventionist and was partly based on the DSA findings. Given the abundant perforators located in the vertebrobasilar system and in the interest of accomplishing excellent angiographic outcomes, we used the smallest number of stents possible to decrease the risk of postoperative ischemic events. If the lesions were very long, we used a telescopic technique with multiple stents. All patients underwent the placement procedures of PED or conventional stent under general anesthesia and *via* a transfemoral approach, and systemic heparinization was administered after placement of the sheath. A suitable guiding catheter was navigated to the C1–C2 level of the vertebral artery. Three-dimensional rotational DSA was performed to choose the best working projection and to measure the parameters of the aneurysms and diameter of the parent artery. Suitable stents were selected according to the aneurysm measurements. Moreover, if aneurysmal or large eccentric lumens originated from the lesions and a stent alone provided an unsatisfactory angiographic outcome with a high recurrence rate, we used the jailing technique to coil the aneurysmal and large eccentric lumens with the assistance of stents.

### Statistical Analysis

Statistical analyses were performed using SPSS 24.0 (IBM Corp., Armonk, NY, United States). According to univariable analyses, between-group comparisons were performed by the *t* test for numeric variables and the χ^2^ test for categorical variables. Multivariable logistic regressions were performed to identify the independent associations between candidate predictor variables and aneurysm occlusion after endovascular treatment between the PED group and control group. Statistical significance for the analyses was defined as *P* < 0.05.

## Results

### Patient and Aneurysm Characteristics

This case series included 11 female patients (14.1%) and 67 male patients (85.9%) (female: male ratio, 1.0:6.1) [age range, 10–71 years; mean ± standard deviation (SD), 49.3 ± 12.6 years]. According to the patients’ major symptoms and preoperative MRI and computed tomography findings, we divided their symptoms into five categories: incidental symptoms, non-specific symptoms, stroke, SAH, and mass effect. For the diagnosis of presentation from mass effect, first, we excepted the stroke and SAH according to CT and MRI; second, according to MRI, the conspicuous compression of brain stem was observed in patients confirmed by preoperational MRI; third, the presentations of patients were associated with the neurological deficit of posterior cranial nerves. The patient demographics and aneurysm characteristics in the PED group and control group are summarized in Table 1.

**TABLE 1:** Patient demographics and aneurysm characteristics.

	**No./Ave (range)**		**%/Stdev**			
			
	**Treatment group**	**Control group**	**Treatment group**	**Control group**	**OR (95% confidence interval)**	***P* Value**
Patients	42	36				
Age	47.9 (10–71)	50.6 (28–69)	±15.0	±9.4	1.017 (0.981–1.055)	0.355
Female sex	8	3	19.9%	8.3%	1.545 (0.242–9.850)	0.353
Hypertension	19	19	45.2%	52.8%	1.289 (0.522–3.186)	0.582
Diabetes	3	2	7.1%	5.6%	0.608 (0.091–4.049)	0.607
Presentation						0.425
Incidental	7	10	16.7%	27.8%		
Non-specific symptoms^∗^	8	1	19.0%	2.8%		
Stroke	7	11	16.7%	30.6%		
SAH	1	3	2.4%	8.2%		
Mass effect	19	11	45.2%	30.6%		
Aneurysms	44	39				
Size (mm)						
Large (10–25)	36	28	81.8%	71.8%	1.341 (0.481–3.742)	0.575
Giant (>25) VBD^	9 7	10 12	18.2% 15.9%	28.2% 30.8%	2.349 (0.817–6.753)	0.113
Location						0.893
LVA	16	14	36.4%	35.9%		
RVA	21	20	47.7%	51.3%		
BA	5	5	11.4%	12.8%		
VBJ	2	0	4.5%	0%		
Branch^#^						0.320
AICA	4	2	9.1%	5.1%		
PICA	21	11	47.7%	28.2%		
VA	1	0	2.3%	0%		
NO	18	26	40.9%	66.7%		
Therapy modality						
Stents & alone	31	11	70.5%	28.2%		
Stents & +coils	13	28	29.5%	71.8%	0.165 (0.064–0.427)	<0.001
Number of devices implanted	52 (1.2,1–4)	75 (1.9,1–4)				<0.001
1	38	14	86.4%	35.9%		
2	5	16	11.4%	41.0%		
3	0	7	0%	18.0%		
4	1	2	2.2%	5.1%		

*^∗^The symptoms without association with lesions confirmed by computerized tomography (CT) and/or magnetic resonance imaging (MRI); VBD^, vertebrobasilar dolichoectatic aneurysms.^#^The branch arteries affected by lesions or covered by stents; &Stents refers to PEDs and conventional stents (Neuroform, Enterprise, Solitaire and Lvis).*

### Technical Success and Immediate Angiographic Results

**FIGURE 1:**
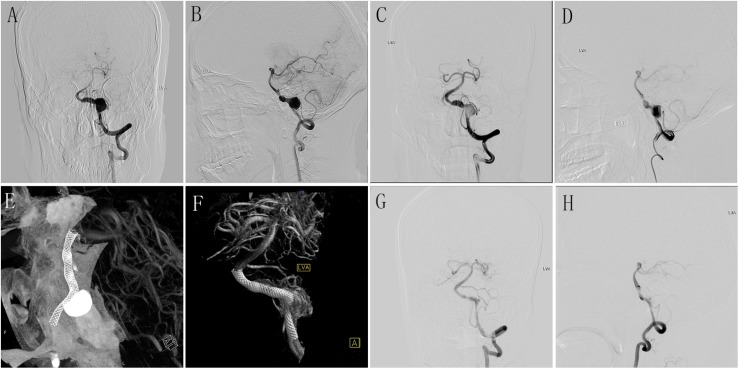
**(A,B)** Preoperative anteroposterior **(A)** and lateral **(B)** DSA of LVA showing a large aneurysm. **(C,D)** Immediately postoperative anteroposterior **(C)** and lateral **(D)** DSA of LVA showing the obvious stasis of contrast agent in aneurysmal lumen. **(E,F)** Postoperative Dyna-CT showing the well wall apposition of the PED (4.5 mm × 35 mm); **(G,H)** DSA follow-up of anteroposterior **(G)** and lateral **(H)** LVA at 6 months after the procedure showing the well reconstruction of diseased vessel. DSA, digital subtracted angiography; LVA, left vertebral artery; PED, pipeline embolization device.

All large or giant VBAs underwent successful endovascular treatment with PEDs or conventional stents. Forty-two patients (53.8%, 42/78) with 44 lesions (53.0%, 44/83) underwent endovascular therapy with PEDs (Figure 1); 36 patients (46.2%, 36/78) with 39 lesions (47%, 39/83) underwent endovascular therapy with conventional stents. After the procedure, we selected the O’Kelly–Marotta grading scale as the standard criterion by which to evaluate the immediate angiographic outcome. Occlusion was graded according to the O’Kelly–Marotta scale as complete occlusion (D), trace filling (C), entry remnant (B), or aneurysm filling (A) (O’Kelly et al., 2010). The evaluation of imaging was conducted by the special group composed of experienced neurointerventionalists. We divided the immediate angiography results into two groups: excellent results (including C and D) and poor results (including A and B). The excellent result rate in the PED group and control group was 27.3% (12/44) and 71.8% (28/39), respectively, with obvious statistical significance [odds ratio (OR), 0.147; 95% confidence interval (CI), 0.056–0.386; *P* < 0.001]. The details of the treatment techniques and immediate angiographic outcomes of the aneurysms are summarized in Tables 1, 2.

**TABLE 2:** Immediate angiographic results and mRS at discharge.

	**No. Ave/(range)**		**%/stdev**			
	**Treatment group**	**Control group**	**Treatment group**	**Control group**	**OR (95% confidence interval)**	***P* Value**
Immediate angiographic results	44	39	100%	100%		
Excellent results	12	28	27.3%	71.8%	0.147 (0.056–0.386)	<0.001
C	9	19	20.5%	48.7%		
D	3	9	6.8%	23.1%		
Poor results	32	11	71.7%	28.2%		
A	23	6	52.3%	15.4%		
B	9	5	19.4%	12.8%		
Clinical outcome at discharge#	42	36	100%	100%		
Excellent clinical outcome	40	35	95.2%	97.2%	0.571 (0.051–6.575)	0.653
Poor clinical outcome	2	1	4.8%	2.8%		
Complications^∗^	4	2	9.5%	5.6%	0.559 (0.096–3.246)	0.517

*^#^We divided clinical outcome at discharge into two group, namely excellent clinical outcome (the mRS score of 0–2) and poor clinical outcome (the mRS score of 3–6). ^∗^The complications of patients during peri-operation.*

### Complications and Clinical Outcomes

The modified Rankin scale (mRS) score at discharge was 0 to 2 in 75 patients (96.2%, 75/78), including 40 patients in the PED group (95.2%, 40/42) and 35 patients in the control group (97.2%, 35/36). We divided the clinical outcomes at discharge into two groups: excellent clinical outcomes (mRS score of 0–2) and poor clinical outcomes (mRS score of 3–6). There was no difference in the rate of excellent clinical outcomes between the two groups (95.2% vs. 97.2%; OR, 0.571; 95% CI, 0.051–6.575; *P* = 0.653). The clinical outcomes at discharge are summarized in Table 2.

**FIGURE 2:**
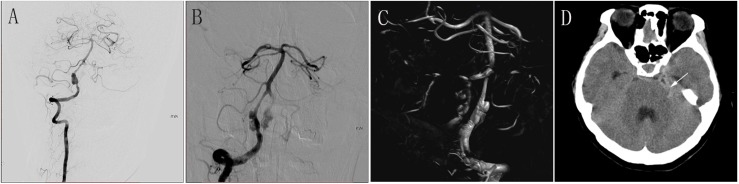
**(A)** Preoperative anteroposterior DSA of the RVA showing a large aneurysm. **(B)** Immediately postoperative DSA of the RVA showing obvious stasis of contrast agent in the aneurysmal lumen. **(C)** Immediately postoperative three-dimensional construction showing good wall apposition of the PED (3.0 mm × 35 mm). **(D)** CT at 1 day after the procedure showing SAH. DSA, digital subtraction angiography; RVA, right vertebral artery; PED, pipeline embolization device; CT, computed tomography; SAH, subarachnoid hemorrhage.

No complications were observed in any patient during the procedure. Postoperative complications occurred in five patients, including three patients in the PED group (7.1%, 3/42) (Figure 2) and two patients in the control group (5.6%, 2/36). No statistical difference in the incidence of complications was confirmed between the two groups (OR, 0.765; 95% CI, 0.121–4.851; *P* = 0.776). Detailed information of complications between PED group and control group was summarized in Table 3.

**TABLE 3:** Complications of patients.

**Number**	**Group**	**Presentations**	**Size (mm)**	**Location**	**Treatment**	**Description**	**Last mRS**
1	PED group	Mass effect	21.2 (LVA)/ 12.0 (RVA)	DVA	2 PED (3.0 mm × 35 mm)	Sudden severe headache 1 day after procedure, SAH confirmed by CT with Hunt-Hess sale of 2	1
2	PED group	Mass effect	23.1	BA	4 PED (3.0 mm × 35 mm)	Died from severe brainstem compression 3 days after procedure	6
3	PED group	Mass effect	25.6	BA	1 PED (3.75 mm × 20 mm) +coils	Died from severe brainstem compression 2 days after procedure	6
4	Control group	Mass effect	34.0	RVA	3 Enterprise (4.5 mm × 37 mm)	Pneumonia	2
5	Control group	Incidental	18.6	RVA	2 Enterprise (4.5 mm × 37 mm) +coils	Sudden severe headache accompanying with nausea and vomit, cerebellar hemorrhage confirmed by CT 2 days after procedure	1

### Imaging and Clinical Follow-Up

#### Angiographic Follow-Up

At least one DSA follow-up was available among 73 patients (93.6%, 73/78) with 78 lesions (94.0%, 78/83) (mean follow-up period, 15.8 ± 12.7 months; range, 6–73 months). In general, we selected the final DSA follow-up of every patient as the time point at which to evaluate the efficacy of PEDs and conventional stents. The complete occlusion rate was 90.2% (37/41) in the PED group and 70.3% (26/37) in the conventional group, with statistical significance (OR, 3.913; 95% CI, 1.122–13.562; *P* = 0.032). The concrete DSA follow-up data are summarized in Table 4.

**TABLE 4:** The results of imaging and clinical follow-up.

	**No. Ave/(range)**		**%/Stdev**			
	**Treatment group**	**Control group**	**Treatment group**	**Control group**	**OR (95% confidence interval)**	***P* Value**
DSA follow-up^∗^	41	37	100%	100%		
A	0	2	0%	5.4%		
B	1	4	2.5%	10.8%		
C	3	5	7.3%	13.5%		
D	37	26	90.2%	70.3%	3.913(1.122–13.652)	0.032
DSA follow up of VBD D DSA follow up of branch arteries Occlusion	7 4 25 4	12 6 13 2	100% 57.1% 100% 16.0%	100% 50% 100% 15.4%	0.750 (0.115–4.898) 0.955 (0.150–6.056)	0.764 0.961
MRI follow-up^&^	15	13	100%	100%		
Reduction	10	3	66.7%	23.1%	6.667 (1.244–35.714)	0.027
Stable	4	1	26.7%	7.7%		
Enlargement	1	9	6.7%	69.2%		
Clinical follow-up^#^	40	36	100%	100%		
Excellent clinical outcome	38	28	97.5%	77.8%	5.429 (1.069–27.556)	0.041
Poor clinical outcome	2	8	2.5%	22.2%		
Clinical follow-up of VBD	7	12	100%	100%		0.019
Excellent clinical outcome	7	8	100%	66.7%		

*^∗^Every patient accepting follow-up underwent more than one time DSA follow-up, and we selected final DSA follow-up as the criterion to evaluate the efficiency of endovascular treatment with PEDs or conventional stents. ^&^According to the results of preoperative MRI, combining with MRI follow-up, we divide the changes of mass effect as three categories, namely reduction, stable, and enlargement. ^#^We divided clinical outcome at discharge into two group, namely excellent clinical outcome (the mRS score of 0–2) and poor clinical outcome (the mRS score of 3–6); VBD, vertebrobasilar dolichoectatic aneurysms.*

#### MRI Follow-Up

**FIGURE 3:**
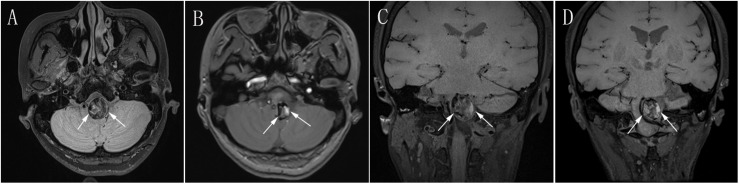
**(A)** Preoperative axial MRI showing a giant mass effect around the brain stem (white arrow). **(B)** Postoperative follow-up axial MRI at 2 years after PED treatment showing obvious reduction of the mass effect (white arrow) compared with preoperative axial MRI in **(A)**. **(C)** Preoperative coronal MRI showing a giant mass effect resulting in severe compression of the brain stem (white arrow). **(D)** Postoperative coronal MRI at 2 years after PED treatment showing obvious reduction of the mass effect (white arrow) compared with preoperative coronal MRI in **(C)**. MRI, magnetic resonance imaging; PED, pipeline embolization device.

With the assistance of preoperative MRI findings, one patient was confirmed to have *de novo* infarction. Forty-five (59.2%, 45/76) patients were diagnosed with large or giant VBAs with a mass effect as confirmed by preoperative MRI. Therefore, the postoperative change in the mass effect was the key factor in evaluation of the efficiency of endovascular treatments in both the PED group and control group. MRI follow-up was available in 28 patients (62.2%, 28/45) (mean follow-up period, 17.8 ± 13.9 months; range, 6–73 months), including 15 patients in the PED group and 13 patients in the control group. By analyzing the results of MRI follow-up and comparing with preoperational MRI by experienced neurointerventionalists, there was a statistical difference in the mass effect reduction rate between the PED group and control group (66.7 vs. 23.1%; OR, 6.667; 95% CI, 1.244–35.714; *P* = 0.027) (Figure 3). The detailed MRI follow-up data are summarized in Table 4.

#### Clinical Follow-Up

Clinical follow-up data were available in 76 patients (97.4%, 76/78) (mean follow-up, 29.3 ± 19.2 months; range, 1–83 months) by enrolling patients and communicating *via* telephone, including 40 patients in the PED group (95.2%, 40/42) and 36 patients in the control group (100.0%, 36/36). According to the clinical follow-up data, the mRS score was 0 to 2 (excellent clinical outcome) in 66 patients (86.8%, 66/76), including 38 patients in the PED group (97.5%, 38/40) and 28 patients in the control group (77.8%, 28/36). In the PED group, one patient’s mRS score worsened to 6 because of the occurrence of SAH as reported by his or her relatives. In the control group, the mRS score was 3 to 6 (poor clinical outcome) in eight patients, including seven patients who died with an mRS score of 6. There was a statistical difference in the rate of an excellent clinical outcome between the PED group and control group (97.5% vs. 77.8%; OR, 5.429; 95% CI, 1.069–27.556; *P* = 0.041). The detailed clinical follow-up outcomes are summarized in Table 4.

## Discussion

### Current Therapeutic Status of VBAs

Vertebrobasilar aneurysms is a catastrophic life-threatening neurovascular disease associated with high morbidity and mortality (Debette et al., 2015). There is no consensus in the management strategies of VBAs (Che et al., 2014; Kühn et al., 2015). Endovascular coiling has lower morbidity but comparatively inferior occlusion outcomes (Fargen et al., 2013). This poor treatment condition for VBAs created an impetus toward elective treatment (PEDs), aiming at a redirection of blood flow along the parent vessel. The expected effects are induced thrombosis of the aneurysmal segment of the vessel and vessel remodeling by hemodynamical change (Sebastian et al., 2014). In a recent study, preliminary experience in the treatment of intradural vertebral artery aneurysms by PEDs showed satisfying short-term results (Kühn et al., 2015). As others have observed, aneurysms located in the basilar artery can be cured with single-modality flow diversion, particularly for the aneurysms with relatively small sizes (Natarajan et al., 2015; Bhogal et al., 2017). However, whether the safety and efficacy of PEDs are better than those of conventional stents for the treatment of large or giant non-saccular VBAs with a relatively poor natural history remains unclear.

### Conventional Endovascular Treatment of VBAs

Endovascular treatment modalities can be divided into reconstructive techniques and deconstructive techniques. The harm secondary to artery occlusion arising from the aneurysm is completely incalculable, which further limits the use of deconstructive techniques (Sebastian et al., 2014). Additionally, aneurysms can grow contralaterally after occluding a vertebral artery aneurysm (Katsuno et al., 2009). For the aneurysms located in the basilar artery, dominant vertebral artery, or major branches, reconstructive approaches are more frequently chosen (Fang et al., 2018). The function of reconstructive techniques have been confirmed, aiming to alter the intra-aneurysmal flow dynamics and thus promote the formation of thrombus within the aneurysm and even improve the flow in the parent vessel (Wakhloo et al., 2008). The medical community has gradually recognized that remodeling of cerebral blood flow is beneficial and more appropriate than aneurysm sac occlusion (Yeung et al., 2012). Conventional stents were designed to be used primarily as an adjunctive treatment to provide structural support for coil embolization of cerebral aneurysms initially (Jorge Arturo et al., 2008; Wakhloo et al., 2008; Yeung et al., 2012). The degree of intra-aneurysmal flow reduction achieved with conventional stents is usually too low to complete thrombosis for aneurysms after treatment (Wakhloo et al., 2008; Sebastian et al., 2014). Studies indicate that reduced stent porosity promotes favorable flow modification (Zang et al., 2015). In theory, the metal coverage of conventional stents can be increased by the overlapping technique, accomplishing excellent flow diversion in the aneurysmal lumen. In fact, Kojima et al. (2013) reported that the reduction of velocity within the aneurysm after the treatment of multiple conventional stents was not as conspicuous as with the flow diverter.

### PED Technique in Treating VBAs

With the enhancement of endovascular materials, the PED offers approximately 30% to 35% coverage of the aneurysm neck by its metallic struts. The high density of coverage is designed to alter flow and induce aneurysm occlusion even without intrasaccular coils (Kallmes et al., 2009). The PED is feasible in treating some aneurysms located in posterior circulation that are difficult or impossible to treat with standard endovascular techniques (Phillips et al., 2012; Nohra et al., 2013; Bender et al., 2018). In a recent study, the highest complete occlusion rate after PED treatment was found in dissecting aneurysms, followed by saccular and fusiform aneurysms (Griessenauer et al., 2018). This device is designed to initially reduce the intra-aneurysmal flow, leading to aneurysm thrombosis and sealing off the aneurysm from the circulation gradually by inducing neointimal coverage of the PED surface at the aneurysm neck (Szikora et al., 2015). At the same time, reconstruction of the parent artery is achieved with this promising device that maintains blood flow through the parent artery, major side branches, and perforators (Kühn et al., 2015). Adeeb et al. (2018) reported a low incidence of branch occlusion following PED coverage in most vessels; moreover, there was no significant increase in the incidence of ischemic complications secondary to branch occlusion compared with covered branches that remained patent. Therefore, the safety of PEDs in treating VBAs is being confirmed increasingly more frequently. With regard to treatment efficacy, as some recent studies about PEDs, the PED provides durable and complete aneurysm closure rate from 86 to 90% (Phillips et al., 2012; Nohra et al., 2013; Griessenauer et al., 2018).

### Explanation of Our Results

In the present study, adjunctive coils were used less frequently in the PED group than control group. This result is not unexpected because of the different designs of PEDs and conventional stents (Jorge Arturo et al., 2008; Wakhloo et al., 2008; Yeung et al., 2012; Sebastian et al., 2014). The complication rate was not significantly different between the PED group and control group, although the PED group had a higher complication rate than the control group. The PED technique is a promising and safe treatment option for VBAs (Sebastian et al., 2014; Kühn et al., 2015). In the present study, the complete occlusion rate was significantly higher in the PED group than that in the control group, according to the DSA follow-up data. Preoperatively, 39 branch arteries were affected by lesions or stents, including 26 branch arteries in the PED group and 13 branch arteries in the control group. During DSA follow-up, we found that four branch arteries (16.0%, 4/25) were occluded completely in the PED group, except one patient who died of severe brain stem compression at 1 day after the procedure; and two branch arteries (15.4%, 2/13) were occluded completely in the control group. There was no statistical significance for the occlusion rates of branch arteries in the PED group and the control group (16.0 vs. 15.4%; OR, 0.955; 95% CI, 0.150–6.056; *P* = 0.961). Fortunately, the patients whose branch arteries were affected were not conspicuous neurological deficit (mRS: 0–2) during clinical follow-up either in the PED group or in the control group.

However, vertebrobasilar dolichoectasia (VBD), as a specific type of VBAs, is a challenging lesion without an ideal treatment modality, and it usually carries a poor prognosis. The estimated 5-year mortality of patients with VBD is 36.2% (Wolters et al., 2013). With modern endovascular techniques and devices, reconstruction of large and giant dolichoectatic aneurysms is feasible (Van Oel et al., 2012). PEDs provide a promising treatment modality for VBD in patients presenting with either non-compressive symptoms or compressive symptoms (Wang et al., 2019a). In the present study, 19 patients presented with VBD were enrolled into case series, including seven patients in the PED group and 12 patients in the control group. The complete occlusion rate was significantly higher in the PED group than that in the control group (57.1 vs. 50%; OR, 0.750; 95% CI, 0.115–4.898; *P* = 0.764), without any statistical significance, according to the DSA follow-up. At the clinical follow-up, the rate of an excellent clinical outcome (mRS: 0–2) was significantly higher in the PED group than that in the control group (100 vs. 66.7%; *P* = 0.019), with statistical significance.

In some studies, the mass effect was alleviated after PED treatment (Stephan et al., 2013; Mohammad et al., 2017; Wang et al., 2019b). In the present study, the PED group achieved a significantly higher mass effect reduction rate than the control group. At the clinical follow-up, the rate of an excellent clinical outcome was also significantly higher in the PED group than that in the control group. The following elements can contribute to the reduction of thrombosed/cured aneurysms on MRI at follow-up: first, the different design of PED, comparing with conventional stents, is a key element to reduce mass effect. Once intra-aneurysmal thrombus is formed, the size of thrombus would have less possibility to grow because of the isolation of aneurysm from circulation by PED; second, the implantation of adjunctive coils in aneurysmal lumen may limit the reduction of mass effect. Although we do not know the molecular mechanism of the reduction of thrombosed/cured aneurysms clearly, PED technique may be promising in the reduction of thrombosed/cured aneurysms on MRI at follow-up.

## Conclusion

Endovascular treatment of large or giant non-saccular VBAs is challenging. PEDs is a promising and safe treatment modality in treating large and giant non-saccular VBAs and have an acceptable complication rate compared with conventional endovascular treatment. However, long-term clinical and angiographic follow-up is necessary to evaluate the safety and efficacy of these two treatment modalities in treating large or giant non-saccular VBAs.

### Limitations

Our study has several main limitations. First, it was a retrospective study with a relatively small number of patients. Second, it was not a randomized study, which the collection of two groups’ subjects was not undertaken during the same period. Third, the MRI follow-up rate was low, making it difficult to evaluate the effectiveness of the two methods in alleviating the mass effect precisely. A long-term study involving a large cohort is needed to obtain sufficient evidence to support our conclusion.

## Data Availability Statement

The raw data supporting the conclusions of this manuscript will be made available by the authors, without undue reservation, to any qualified researcher.

## Ethics Statement

The protocol of this study was approved by the Beijing Tiantan Hospital’s Institutional Review Board, and performance of the study was approved by the ethics committee of Beijing Tiantan Hospital.

## Author Contributions

JW performed the manuscript writing. LJ and ZW acquired the data. ZD analyzed and interpreted the data. YZ checked the manuscript. ML and XY conceived and designed the research, and handled the funding and supervision.

## Conflict of Interest

The authors declare that the research was conducted in the absence of any commercial or financial relationships that could be construed as a potential conflict of interest.
